# Systemic administration of a TLR7 agonist attenuates regulatory T cells by dendritic cell modification and overcomes resistance to PD-L1 blockade therapy

**DOI:** 10.18632/oncotarget.24327

**Published:** 2018-01-27

**Authors:** Naoto Nishii, Hidetake Tachinami, Yuta Kondo, Yulong Xia, Yoshihisa Kashima, Tatsukuni Ohno, Shigenori Nagai, Lixin Li, Walter Lau, Hiroyuki Harada, Miyuki Azuma

**Affiliations:** ^1^ Department of Molecular Immunology, Graduate School of Medical and Dental Sciences, Tokyo Medical and Dental University (TMDU), Tokyo, Japan; ^2^ Department of Oral and Maxillofacial Surgery, Graduate School of Medical and Dental Sciences, Tokyo Medical and Dental University (TMDU), Tokyo, Japan; ^3^ Birdie Biopharmaceuticals Inc., Iselin, NJ, USA

**Keywords:** TLR7 agonist, resiquimod, immune checkpoint blockade, companion drug, regulatory T cells

## Abstract

Research on immune checkpoint blockade therapy has made great progress in cancer immunotherapy, but the number of patients who benefit from this therapy remains limited. In this study, we examined the effects of monotherapy with systemic low-dose resiquimod, a synthesized TLR7 agonist, and examined its combined effects with PD-L1 blockade in two PD-L1 blockade-resistant tumor models (SCCVII and Colon 26). Resiquimod monotherapy in SCCVII tumors, representing impaired CD8^+^ T cell function and accelerated regulatory T cells (Tregs) within the tumors, efficiently reduced tumor growth with more recruitment of CD8^+^ T cells and a reduction of Treg. The results of resiquimod monotherapy in Colon 26, representing impaired Treg recruitment, were inferior to that in SCCVII. Combined resiquimod treatment with PD-L1 blockade exerted clear additional effects, as it was associated with reduced tumor size, attenuation of Tregs, and an increased ratio of CD8^+^ T cells/Tregs in both tumors. Systemic administration of low-dose resiquimod induced a transient and rapid activation of plasmacytoid and conventional dendritic cells, resulting in enhanced priming of T cells in regional lymph nodes. Experiments with more limited doses of resiquimod that did not yield beneficial effects after single treatment, showed additional effects to PD-L1 blockade and comparable antitumor effects when the frequency of anti-PD-L1 therapy was decreased. Our results suggest that systemic administration of low-dose resiquimod is useful as a companion drug to PD-1/PD-L1 blockade therapy.

## INTRODUCTION

The blockade of immune checkpoint molecules, such as CTLA-4 and PD-1, efficiently enhances antitumor immune responses by eliminating the immunoregulatory function of effector T cells, regulatory T cells (Tregs), myeloid-derived suppressor cells (MDSCs), and tumor-associated macrophages (TAMs) [1–4]. However, individual patients who receive the benefits of immune checkpoint blockade differ greatly by clinical grade and tumor tissue type. Head and neck cancers (HNCs) do not frequently express the tumor-specific mutated antigens (called “neoantigens”) that elicit T-cell immunoreactivity and influence sensitivity to immune checkpoint blockade therapy [5, 6], and their tumor microenvironment (TME) contains abundant regulatory cells, such as Tregs and MDSCs [7–9]. We recently demonstrated that blockade of either CTLA-4 or PD-1 efficiently inhibited the tumor growth of a murine squamous cell carcinoma (SCC) cell line (SCCVII) in syngeneic mice; however, we failed to eradicate the tumors [10]. The ratio of effector CD8^+^ T cells/Tregs (CD8/Treg) closely associated with the prognosis and outcome of immunotherapy [10, 11].

Toll-like receptors (TLRs) recognize a wide panel of exogenous and endogenous ligands, and the binding of natural and synthetic TLR ligands to TLRs induces multiple cytokine production, resulting in enhanced innate and adaptive immunity [12–14]. Small compounds that are designed to bind TLRs are potential drugs for vaccines and adjuvants in infectious disease and cancer. TLR7 and TLR8, which are expressed on endosomes, have structural similarity and recognize microbial single-stranded RNA (ssRNA). Imiquimod (R837) and resiquimod (R848), which are imidazoquinoline compounds of synthesized ssRNAs, bind TLR7 alone and both TLR7 and TLR8, respectively [15, 16]. Resiquimod does not exert mouse TLR8-mediated activation. TLR7/8-mediated signaling induces the production of NF-kB-mediated proinflammatory cytokines and chemokines, and type I interferon (IFN) by activating antigen-presenting cells (APCs), resulting in the augmentation of effector T cells [12, 17, 18]. Nevertheless, imidazoquinolines have been applied in the clinical setting only in the form of a topical treatment or a locally administrated drug for the treatment of non-melanoma skin cancers and viral skin lesions to avoid TLR tolerance and adverse proinflammatory and proapoptotic effects [19–22].

Recently, systemic or intratumoral administration of TLR7 agonists, such as resiquimod and its related conjugate, has shown to induce efficient tumor regression by inhibiting TAM- or MDSC-mediated immune suppression in murine tumor models [23, 24]. In PD-1 blockade therapy-resistant and immunosuppressive tumors, such as HNC, combined treatment with a TLR7 agonist and PD-1 blockade might be an effective cancer immunotherapy. In this study, we examined the effects of monotherapy of systemic low-dose resiquimod and combined therapy with anti-PD-L1 treatment in two PD-L1 blockade-resistant tumor models.

## RESULTS

### The TME of SCCVII presents a more immunosuppressed status

**Figure 1:**
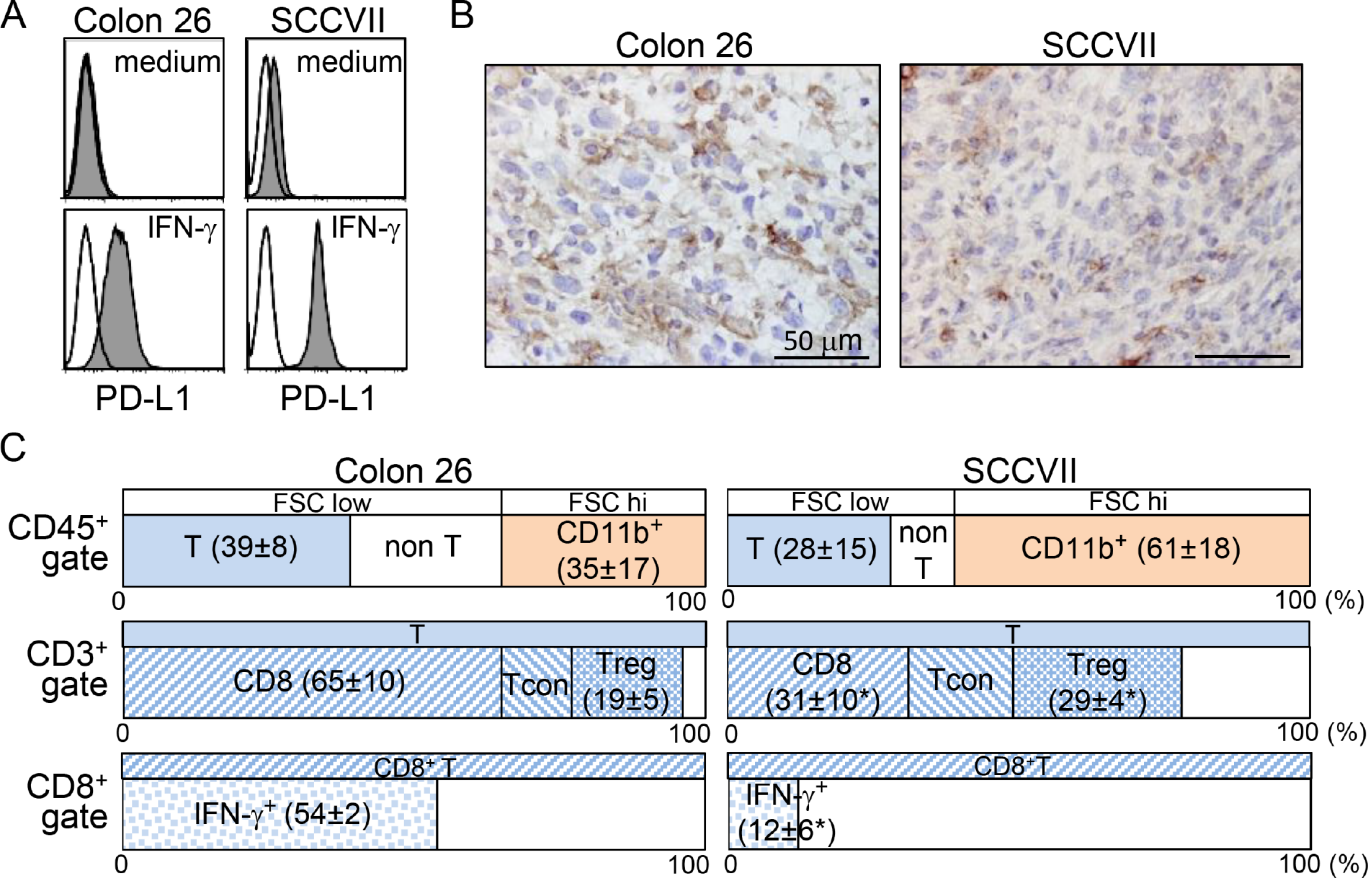
Colon 26 and SCCVII tumors exhibit distinct host immune responses in the TME (**A**) Colon 26 and SCCVII tumor cells lines cultured in the presence or absence of IFN-γ for 72 hr were stained with PE-conjugated control rat IgG2a or anti-PD-L1 (MIH5) mAb. Expression levels of PD-L1 are displayed as shaded histograms with the control staining displayed as open histograms. (**B**) Tumor tissue sections at 20 days after tumor inoculation were stained with anti-PD-L1 (MIH6) mAb. Scale bars = 50 µM. (**C**) TIL fractions from Colon 26 and SCCVII tumor masses at day 19 were stained and analyzed by flow cytometry. To avoid the loss of adherent cells, the percentage of CD45^+^ cells was obtained from cells without *in vitro* stimulation culture. An electronic gate was placed on CD45^+^FSC^low-high^ lymphocytes, and then the proportions of FSC^low^CD3^+^CD11b^-^ (T) and FSC^med-high^CD3^-^CD11b^+^ myeloid cells within CD45^+^ pan-lymphocytes were then analyzed. For T-cell analysis, electronic gates were placed on FSC^low^CD45^+^CD3^+^ (CD3^+^ T) or FSC^low^CD3^+^CD8^+^ (CD8^+^ T) cells, and then the proportions of CD8^+^CD4^-^ (CD8^+^ T), CD8^-^CD4^+^Foxp3^-^ (conventional T, Tcon), CD8^-^CD4^+^Foxp3^+^ (Treg) within T cells, and IFN-γ^+^ cells within CD8^+^ T cells were analyzed. The values show the mean ± SD from each group of five mice. ^*^Statistically different (*p* < 0.05).

Colon 26 is a carcinogen-induced undifferentiated colon carcinoma cell line that originated from BALB/c mice, and SCCVII is a spontaneously arising SCC cell line derived from C3H/HeN (C3H) mice [25, 26]. PD-1:PD-L1 blockade therapy in these tumor models did not completely eradicate the tumors in syngeneic mice ([10] and data discussed below). Although both cultured tumor cell lines showed no or marginal PD-L1 expression, IFN-γ treatment for 72 hr efficiently increased its expression (Figure 1A). Furthermore, immunohistochemistry showed that subcutaneously inoculated tissue tumor cells expressed PD-L1; however, its expression levels varied by lesions (Figure 1B). PD-L1 expression in both tumors is inducible *in vitro* and *in vivo.* To investigate the differences between the host immune responses in both tumors, we examined the phenotypes and activation status of tumor infiltrating lymphocytes (TILs). Compared with Colon 26, CD45^+^ pan-leukocytes in the SCCVII-TIL fractions contained a lower percentage of CD3^+^ T and a greater percentage of CD11b^+^ myeloid cells (Figure 1C). These CD11b^+^ cells presented with high forward scatter, and antigenic profiles that were Gr-1-negative and F4/80-positive, indicating a phenotype consistent with TAMs (Supplementary Figure 4A). CD3^+^ T cells in SCCVII-TILs had a significantly lower percentage of CD8^+^ T and a greater percentage of CD4^+^Foxp3^+^ Tregs. Furthermore, a significantly lower proportion of CD8^+^ T cells expressed IFN-γ. These results show that the TME in SCCVII had impaired effector cytotoxic T lymphocytes (CTLs) and a high recruitment of immunoregulatory TAMs and Tregs.

### Systemic low-dose resiquimod administration induces a transient upregulation of serum IFN-α

To avoid unwanted proinflammatory cytokine production by systemic resiquimod injection, we first used a low dose (1.7 μg/mouse) of resiquimod compared with the dose of other imidazoquinoline-like molecules injected in mice [23, 27]. Serum proinflammatory cytokines in intact and SCCVII tumor-inoculated C3H mice were measured. At 3 hr after resiquimod injection, serum IFN-α increased transiently, but it returned to an undetectable level at 12 hr (Table 1). Similar to IFN-α, IL-6 also increased at 3 hr, albeit only slightly, and was undetectable at 12 hr. This dose of resiquimod did not increase IL-1β and TNF-α. Tumor inoculation slightly upregulated serum IFN-α and IL-6 production, but these diminished quickly. These results indicate that systemic injection of a low-dose of resiquimod did not induce the production of multiple proinflammatory cytokines, but induced temporal activation of plasmacytoid DCs (pDCs), that expressed high levels of TLR7 [28], which may contribute to the selective and transient upregulation of IFN-α.

**Table 1:** Serum cytokines after resiquimod injection

cytokines		IFN-α (pg/ml)		IL-1β (pg/ml)		IL-6 (pg/ml)		TNF-α (pg/ml)	
tumor inoculation*		–	+	+	–	–	+	–	+
hr after resiquimod injection	0	<62	<62	<62	<62	<62	<62	<62	<62
	3	2216 ± 697	3271 ± 2615	<62	<62	138 ± 45	157 ± 74	<62	<62
	12	<62	<62	<62	<62	<62	<62	<62	<62
	24	<62	106 ± 92	<62	<62	<62	<62	<62	<62

^*^40 Gy-irradiated-SCCVII cells.

### Systemic low-dose resiquimod administration efficiently reduces tumor growth and enhances the efficacy of PD-L1 blockade therapy

**Figure 2:**
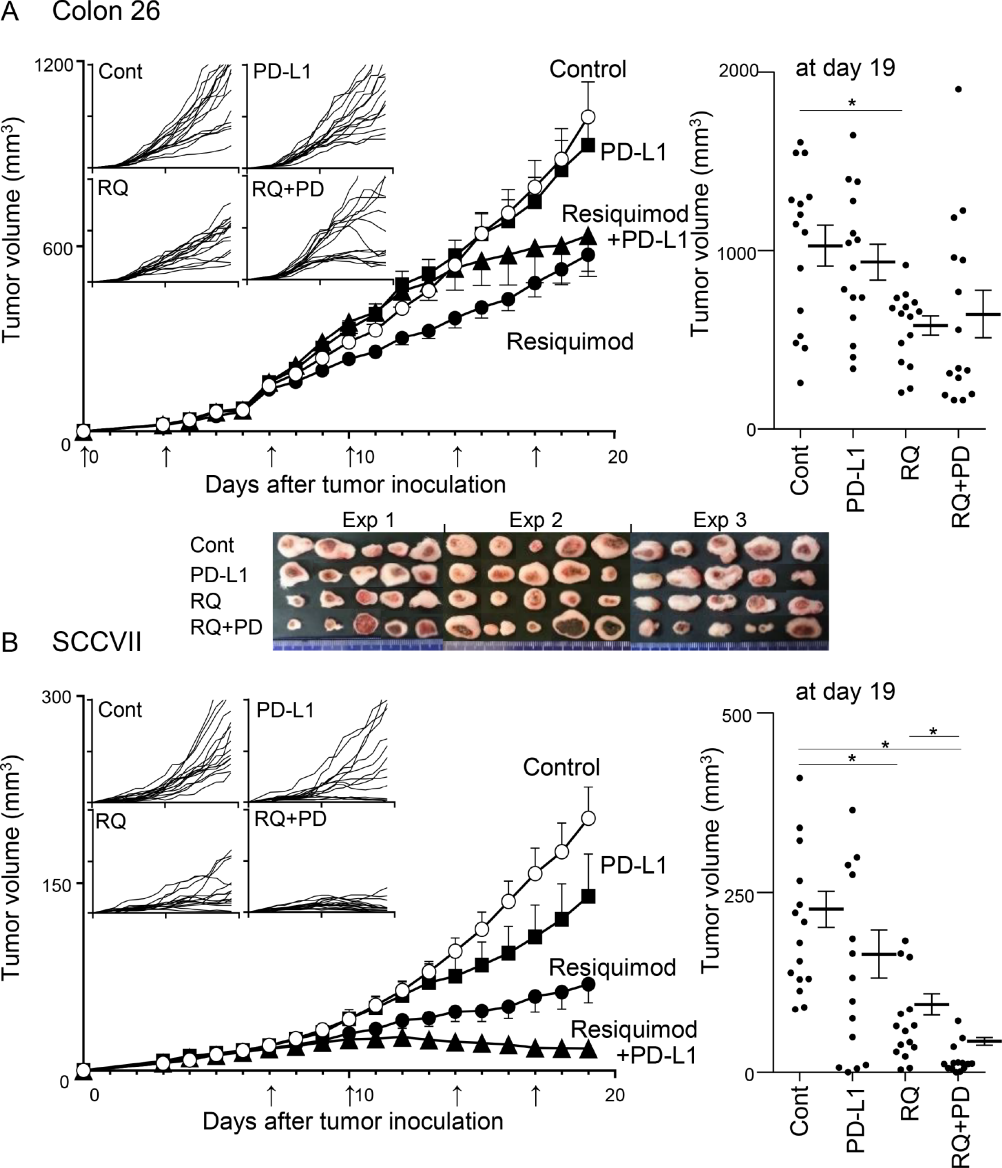
Effects of treatments with resiquimod and/or PD-L1 blockade in Colon 26 and SCCVII tumor models Colon 26 (**A**) and SCCVII (**B**) tumor cells were inoculated into BALB/c and C3H mice, and treatments were started on day 0 and day 7, respectively. Control reagents (PBS or rat IgG), resiquimod (RQ), anti-PD-L1 mAb (PD-L1) and both (RQ+PD) were injected i.p. twice a week until day 17. The arrows indicate the time of treatment. Tumor volume was measured every day. The values are the mean ± SEM from three independent experiments with each group of five mice. ^*^Statistically different (*p* < 0.05). The four smaller panels on the left show the individual growth curves. The final tumor volumes on day 19 are shown in the right panels. Digital photo images of resected Colon 26 tumor masses on day 19 are shown in the lower panel of A.

We examined the effects of monotherapy with either PD-L1 blockade or resiquimod, and of combined therapy in two tumor models (Colon 26 and SCCVII). The addition of resiquimod to each tumor cell culture did not clearly affect tumor growth, indicating that both tumor cells did not directly respond to resiquimod. In preliminary experiments, treatment started at day 0 (early treatment) and day 7 (delayed treatment). Although the delayed treatment in SCCVII tumors showed effects comparable to those of the early treatment, the delayed treatment worsened the effects in Colon 26. Thus, thereafter, we used only early treatment for Colon 26 and delayed treatment for SCCVII. Treatment with anti-PD-L1 monotherapy did not clearly affect the tumor growth of Colon 26 (Figure 2A). A similar treatment in SCCVII slightly decreased the tumor volume, and the growth curves of individual SCCVII tumors reflected three response groups (resistant, moderate, and sensitive) (Figure 2B). Resiquimod monotherapy markedly inhibited tumor growth in both tumor models, and its inhibitory effect was much more obvious in SCCVII. Regarding Colon 26 tumors, an assessment of mean tumor volume did not show combined effects, but individual growth curves showed bilateral groups of tumor size (large and small) (Figure 2A). It should be noted that the images of the resected tumors in the combined therapy showed clear and widespread necrotic lesions with a dark red color, especially in the large tumor masses (Figure 2A). This suggests additional antitumor effects in Colon 26 by the combined therapy. In SCCVII tumors, the combined therapy gradually decreased tumor growth from day 13 to the final observation day (day 19), and the mean tumor volume on the final day was significantly reduced (Figure 2B). The tumor size in over half the mice remained unchanged in the last 4 days, and five mice showed only scar-like lesions, suggesting almost complete eradication of the tumors. Our results demonstrate that systemic low-dose resiquimod administration efficiently reduced tumor growth, and combined treatment with PD-L1 blockade exerted additional effects in two tumor models.

### Resiquimod combined with PD-L1 blockade modulates recruitment of CD8^+^ T cells and Tregs in the TME

**Figure 3:**
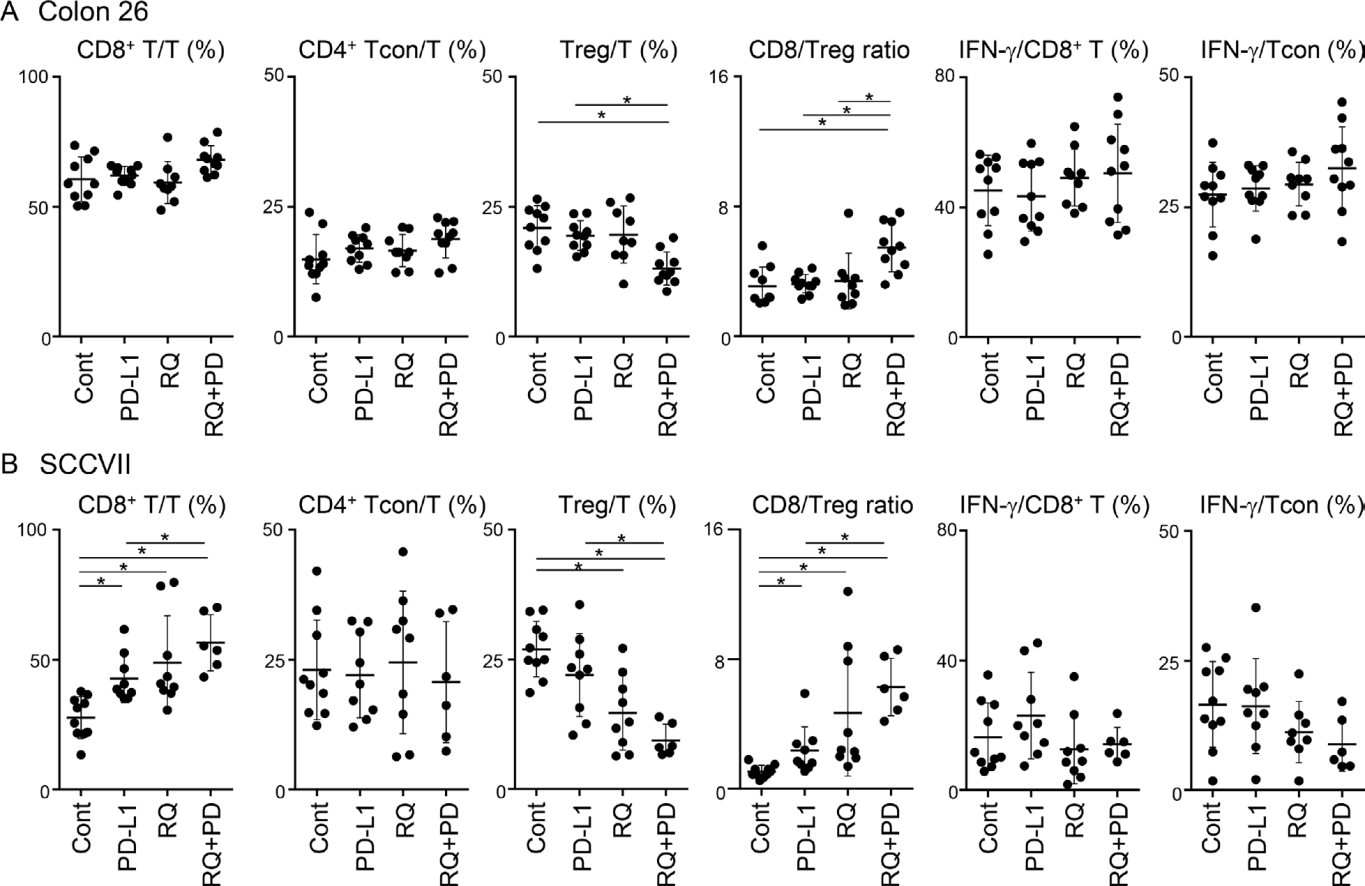
Changes of T-cell subsets and IFN-γ expression in TILs by treatments Tumor masses from Colon 26- (**A**) and SCCVII- (**B**) inoculated mice were collected and TILs were obtained. Cells were stimulated with PMA, ionomycin, and brefeldin A for 6 hr, and then cell surface and intracellular staining was performed. Cells were stained with FITC-anti-CD45, APC-eFluor780-anti-CD3, PE-Cy7-anti-CD4, PerCP-Cy5.5-anti-CD8, APC-anti-Foxp3, and PE-anti-IFN-γ mAbs or appropriate fluorochrome-conjugated control Abs, and then cells were analyzed by flow cytometry. Electric gates were placed on CD45^+^CD3^+^ lymphocytes (T cells), CD45^+^CD3^+^CD8^+^ (CD8^+^ T cells), or CD45^+^CD3^+^CD4^+^Foxp^-^ (Tcon), and the percentages of CD8^+^, Foxp3^-^CD4^+^ (Tcon), and Foxp3^+^CD4^+^ (Treg) within T-cell gate and IFN-γ^+^ cells within CD8^+^ T cells or Tcon were analyzed. Data shown are the combined results from two independent experiments with each group of five mice. The bars show the mean values ± SD. ^*^Statistically different (*p* < 0.05).

The proportions of three T-cell subsets; CD8^+^ T, Foxp3^-^CD4^+^ conventional CD4^+^ T (Tcon), and Foxp3^+^CD4^+^ Treg, within CD45^+^ TILs and IFN-γ expression within CD8^+^ and CD4^+^ Tcon cells were determined on the final day. In Colon 26 tumors, no T-cell subset was clearly affected by either monotherapy, but the combined treatment significantly decreased the Treg proportion compared with the control and PD-L1 blockade groups (Figure 3A). This resulted in an increase in the CD8/Treg ratio. IFN-γ expression levels in both CD8^+^ T and Tcon were increased by the combined treatment, although the difference was not statistically significant. Interestingly, resiquimod monotherapy in SCCVII efficiently increased the proportion of CD8^+^ T cells and decreased the proportion of Tregs, resulting in a marked elevation of the CD8/Treg ratio in some mice (Figure 3B). PD-L1 blockade alone also significantly increased the percentage of CD8^+^ T cells and the CD8/Treg ratio. An additional resiquimod treatment combined with PD-L1 blockade further decreased the proportion of Tregs and resulted in an improved CD8/Treg ratio in most mice. Consistent with the findings in Colon 26 tumors, the expression levels of IFN-γ in both CD8^+^ T and Tcon were not clearly affected. These results indicate that systemic administration of low-dose resiquimod in the Treg-dominant SCCVII markedly decreased the proportion of Tregs and reciprocally promoted CTL recruitment in the TME. Unlike the findings for SCCVII, similar treatment in a relatively CTL-rich Colon 26 tumor model reduced Treg recruitment without affecting CTLs. Taken together, our results indicate that systemic low-dose resiquimod administration in addition to PD-L1 blockade is especially effective for elevating the CD8/Treg ratio in the TME.

### Resiquimod quickly enhances activation of two types of DCs

**Figure 4:**
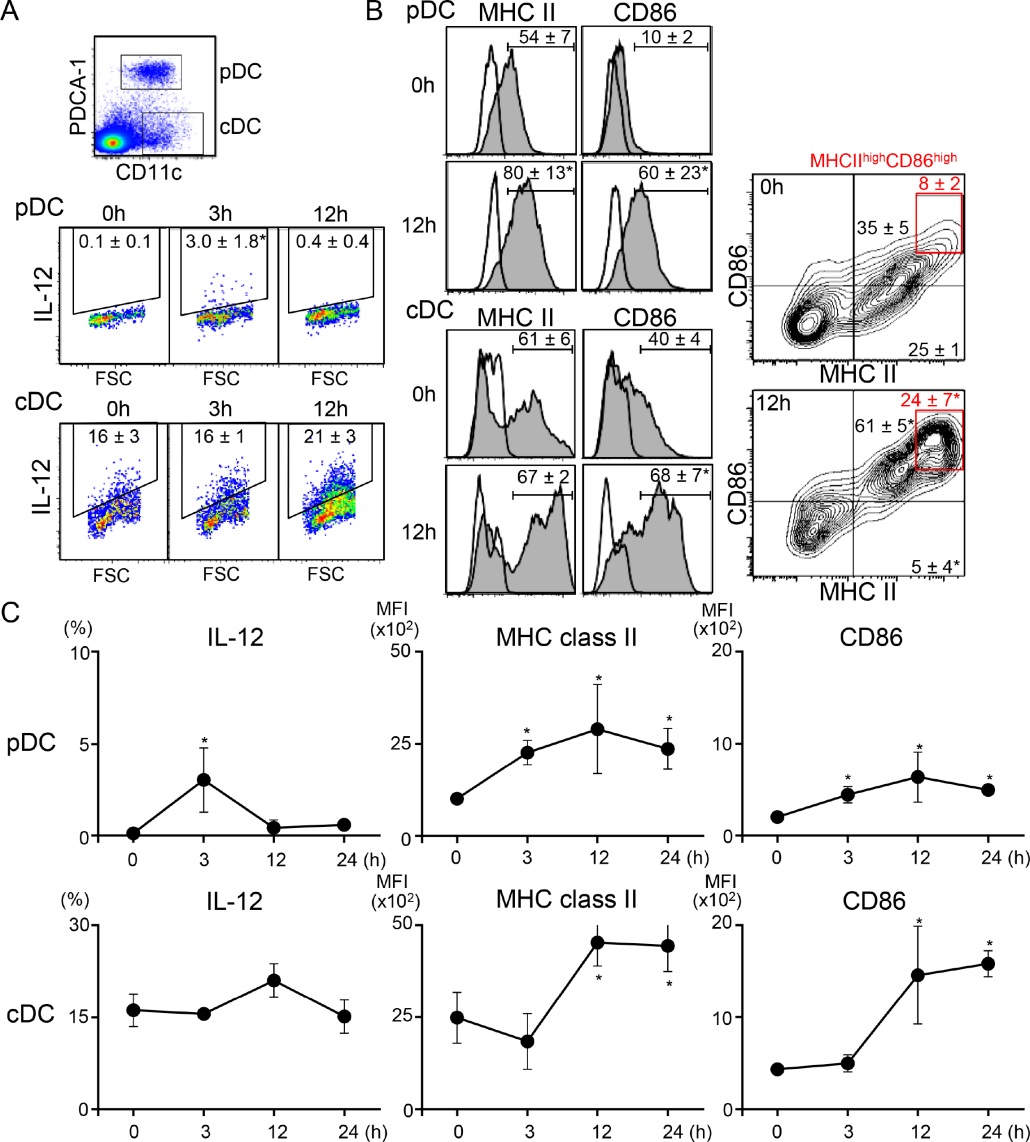
Systemic administration of low-dose resiquimod induces activation of both types of DCs RLN cells from irradiated SCCVII tumor-injected mice at the indicated time points were isolated. Resiquimod was i.p. injected at the time of tumor injection. Cells were stained with PerCP-Cy5.5-anti-CD11c, APC-anti-PDCA-1, FITC-anti-MHC class II, and either PE-anti-CD86 or PE-anti-IL-12 mAbs or appropriate fluorochrome-conjugated control Abs. Stained cells were analyzed by flow cytometry. An electronic gate was placed on PDCA-1^+^CD11c^+^ for pDCs or PDCA-1^-^CD11c^+^ for cDCs, as shown in the upper panel of A, and the expression levels of IL-12, MHC class II, and CD86 were analyzed. Expression levels of IL-12 (**A**) and MHC class II and CD86 (**B**) at the indicated time points are presented as dotted plots, histograms, or contour plots. The values shown are the mean percentages ± SD in each DC fraction. The red boxes in (B) show MHC class II^high^CD86^high^ fractions and their mean values are shown in red. (**C**) Kinetic changes in the percentage of IL-12^+^ cells and the mean fluorescence intensity (MFI) of MHC class II and CD86 expression in each DC fraction are shown. All values shown are the mean ± SD from three mice. Representative data are shown. Statistically different from the values at 0 hr (*p* < 0.05).

To understand the mechanisms by which systemic low-dose resiquimod administration enhanced antitumor responses by modulating Tregs and CD8^+^ T cells, we examined much earlier time points after resiquimod injection, focusing on DC function. We showed that serum IFN-α was transiently upregulated at 3 hr after resiquimod injection (Table 1); thus, DCs, especially pDCs, might have been targeted by resiquimod, as pDCs express high levels of TLR7 [28]. We examined the expression of IL-12, MHC class II, and CD86 in PDCA-1^+^ pDCs and PDCA-1^-^ conventional DCs (cDCs) in regional lymph nodes (RLNs). As expected, resiquimod application induced IL-12 expression by pDCs at 3 hr. IL-12 expression then declined at 12 hr (Figure 4A and 4C), with kinetical change similar to that of IFN-α as shown in Table 1. Cell surface MHC class II and CD86 expression, which are critical for antigen-presenting capacity, were also increased on pDCs, with peak expression at 12 hr (Figure 4B and 4C). In the case of cDCs, transient upregulation of IL-12 was a little delayed (at 12 hr), and there was a simultaneous upregulation in MHC class II and CD86 expression. In particular, MHC class II^high^CD86^high^ fraction in cDCs was dramatically increased after 12 hr (Figure 4B, right panels). The upregulated expression of MHC class II and CD86 were maintained until 24 hr. To examine whether cDC activation by resiquimod is dependent on pDC, we compared IL-12 expression by cDCs *in vitro* using whole and pDC-depleted splenocytes. Even after depletion of pDCs, resiquimod stimulation induced a comparable level of IL-12 expression (Supplementary Figure 1), suggesting that resiquimod can directly activate cDCs without pDC-mediated help. These results indicate that systemic resiquimod administration augmented the activation of both pDCs and cDCs in the circulation and RLNs.

### Resiquimod enhances activation of CD8^+^ T cells in RLNs and the TME

**Figure 5:**
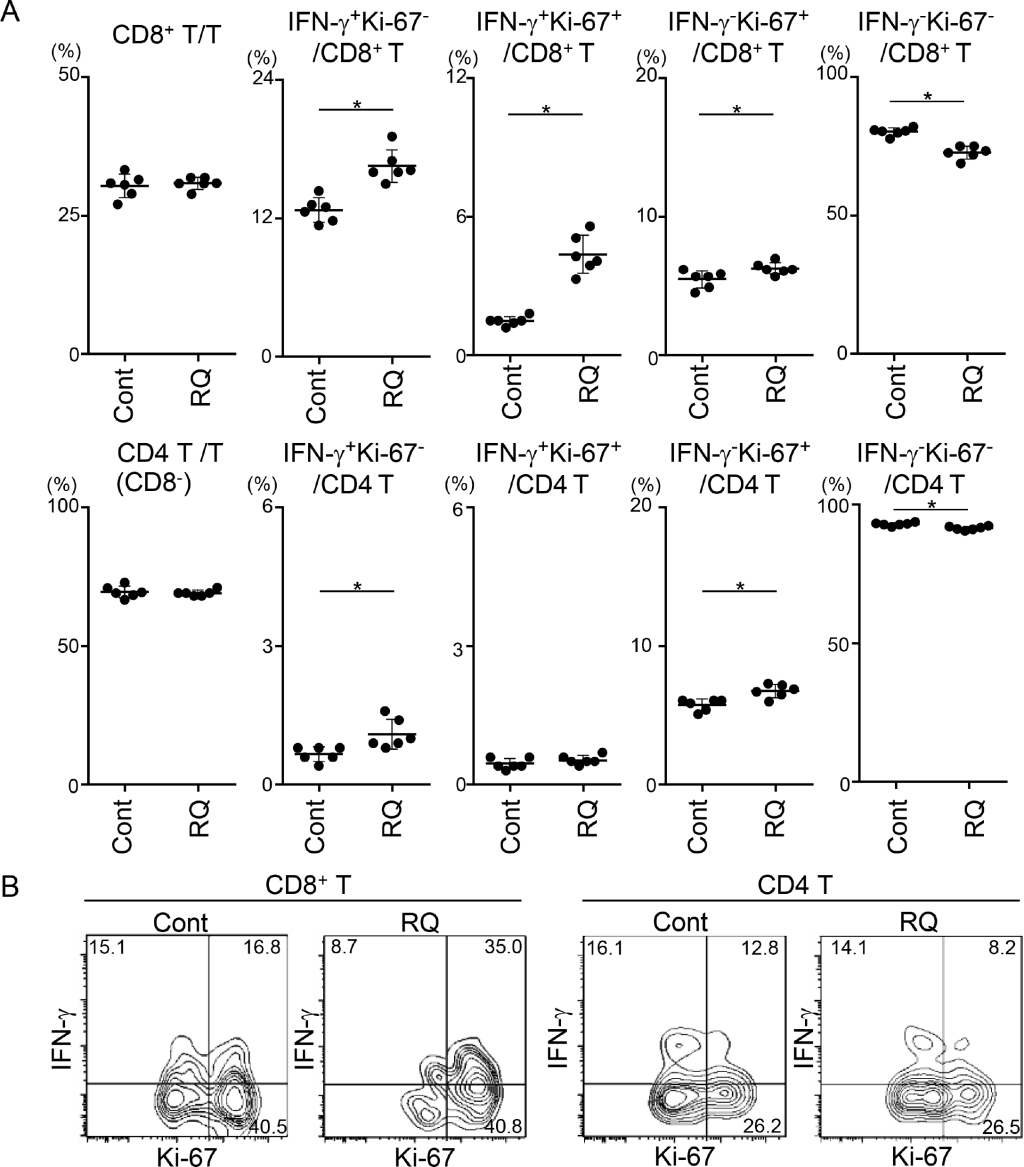
Resiquimod enhances activation of CD8^+^ T cells in RLNs and the TME RLNs (**A**) and tumor masses (**B**) from SCCVII-inoculated mice treated with control reagents or resiquimod at days 0, 3, 7, and 10 were obtained at 12 days. Tumor masses from five to six mice were combined, and TIL fractions were isolated. RLN cells and TILs were stimulated and stained with Brilliant Violet 510-anti-CD45, APC-eFluor780-anti-CD3, PerCP-Cy5.5-anti-CD8, APC-anti-IFN-γ, FITC-anti-Ki-67 mAbs or appropriate fluorochrome-conjugated control Abs. Stained cells were analyzed by flow cytometry. (A) Electric gates were placed on CD3^+^, CD3^+^CD8^+^, or CD3^+^CD8^-^ (as CD4^+^ T) lymphocytes, and then the percentages of the indicated fractions were analyzed. Data shown are representative from two independent experiments with similar results. The bars show the mean values ± SD from each group of six mice. ^*^Statistically different (*p* < 0.05). (B) An electronic gate was placed on CD45^+^CD3^+^CD8^+^ or CD45^+^CD3^+^CD8^-^ (as CD4^+^ T) lymphocytes, and the expression levels of Ki-67 and IFN-γ are shown as contour plots. Data are representative from two independent experiments with similar results.

Because activation of DCs at an earlier time point might amplify the priming of T cells in RLNs, we next examined the activation status of T cells by evaluating IFN-γ (a critical effector cytokine) and Ki-67 (a proliferation marker) at day 12. Resiquimod administration did not affect the percentage of CD8^+^ T cells, but it significantly increased the proportion of three activated states (IFN-γ^+^Ki-67^-^, IFN-γ^+^Ki-67^+^, and IFN-γ^-^Ki-67^+^) of CD8^+^ T cells and reciprocally decreased the proportion of non-activated IFN-γ^-^Ki-67^-^ CD8^+^ T cells (Figure 5A). In contrast to that of CD8^+^ T cells, activation of CD4^+^ T cells, as assessed by the CD8^-^CD3^+^ fraction, was minimal. Consistent with the findings for RLN CD8^+^ T cells, TIL CD8^+^ T cells, but not TIL CD4^+^ T cells, also showed a greater proportion of the IFN-γ^+^Ki-67^+^ fraction at this time point (Figure 5B). These results suggest that resiquimod administration efficiently enhanced the priming of CD8^+^ T cells through modification of the antigen-presenting capacity of DCs.

### A limited dose of resiquimod exerts combinational effects with PD-L1 blockade therapy

**Figure 6:**
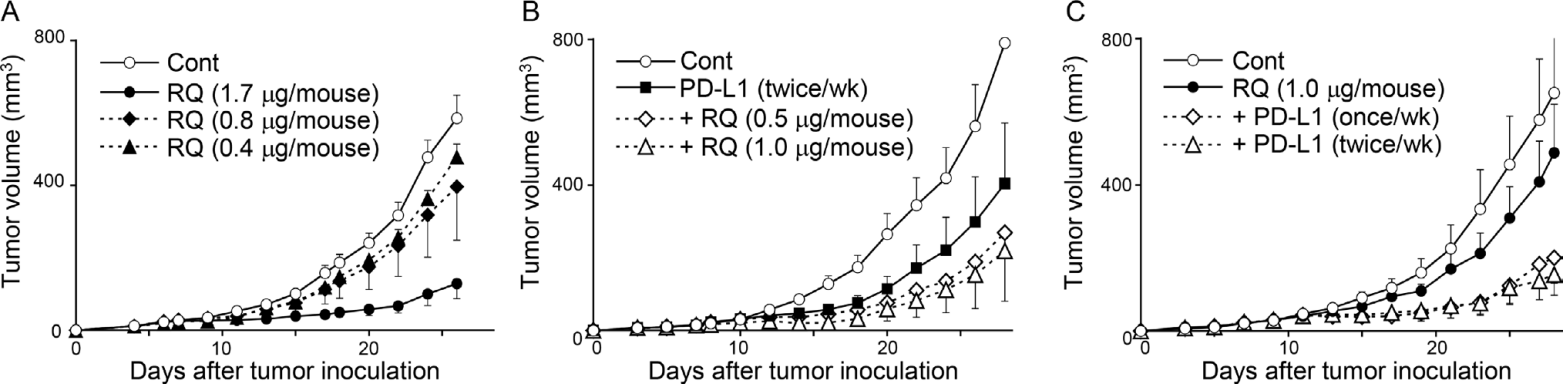
A limited dose of resiquimod exerts combinational effects with PD-L1 blockade therapy C3H mice received s.c. injection of SCCVII tumor cells, and tumor growth was measured as described in Figure 2. The indicated doses of resiquimod (RQ) and anti-PD-L1 mAb (200 μg/mouse) were injected i.p. twice per week unless otherwise noted. All treatments were started at 7 day after tumor inoculation. The values are the mean ± SEM from each group of five mice. Data shown are representative from two independent experiments with similar results.

Our results suggest that resiquimod may be used as a companion drug for PD-1/PD-L1 blockade therapy. We titrated the concentration of resiquimod (0.4, 0.8, and 1.7 μg/mouse) and examined the tumor volume of SCCVII. The inhibitory effects of resiquimod monotherapy were dose-dependent, and doses of 0.8 and 0.4 μg showed marginal effects (Figure 6A). Additionally, we examined the effects of limited doses (1.0 and 0.5 μg/mouse) of resiquimod in addition to the PD-L1 blockade. Although these doses were not effective in monotherapy, additional resiquimod treatment along with the PD-L1 blockade further decreased the tumor volume (Figure 6B). We next examined whether the decreased frequency of anti-PD-L1 therapy was still effective when it was combined with resiquimod. We decreased the frequency of anti-PD-L1 treatment to the half (from twice per week to once per week). The decreased frequency of anti-PD-L1 therapy under the resiquimod treatment consistently showed comparable effects (Figure 6C). These results suggest that limited doses of systemic resiquimod administration enable to overcome resistance to PD-L1 blockade, and allow for the decreased usage of the anti-PD-L1 antibody.

## DISCUSSION

We demonstrated the differential effects of systemic low-dose resiquimod administration in monotherapy and in combined treatment with PD-L1 blockade in two PD-L1 blockade-resistant tumor models that exhibit different profiles of TILs. Resiquimod monotherapy resulted in some tumor reduction in Colon 26, and showed a relatively higher recruitment and activation of CD8^+^ T cells in the TME. Combined treatment did not clearly affect outside tumor size, but it did augment the necrotic lesions of inside tumors that were correlated with a marked reduction of Tregs in the TME. Unlike Colon 26 tumors, resiquimod monotherapy in SCCVII tumors with impaired CD8^+^ T cell activation and accelerated Treg recruitment in the TME markedly decreased tumor size along with CD8^+^ T cell accumulation and a reduction of Tregs. Combined resiquimod treatment with PD-L1 blockade exerted additional effects on tumor size and Treg and CD8^+^ T cell recruitment.

Our results indicate that the efficacy of systemic resiquimod administration is highly superior in Treg-dominant tumors, such as SCCVII. This suggests that low-dose resiquimod administration converts resistance to PD-L1 blockade therapy that is caused by Treg accumulation. We previously reported that Treg depletion in a SCCVII model resulted in almost complete eradication of tumors [29]; thus, Tregs are critical immune regulators that accelerate SCCVII tumor growth. Even in Colon 26, combined treatment decreased Treg accumulation, resulting in expansion of the necrotic area. In both tumor models, resiquimod treatment was efficacious because it resulted in Treg attenuation in the TME. Previous reports using a relatively high dose of TLR7/8 agonists (resiquimod and loxoribin) also demonstrated *de novo* inhibition of Tregs via modulation of DCs and involvement of IL-6 [30, 31]. Resiquimod-treated DCs have been shown to downregulate Foxp3 expression and suppress the function of Tregs [30]. We also observed that generation of induced Tregs was clearly inhibited by the resiquimod-treated DCs in co-culture experiments with RAG2^-/-^DO11.10 CD4^+^ T cells and OVA-pulsed DCs (Supplementary Figure 2). IL-6 production in the pre-DC culture in the presence of OVA and a maturation–cytokine, TNF-α increased almost ten-fold by resiquimod stimulation (the mean ± SD; without resiquimod, 74.7 ± 42.3 pg/ml and with resiquimod, 650.6 ± 44.5 pg/ml). This suggests that resiquimod-treated DCs inhibit Treg generation and IL-6 secreted from resiquimod-activated DCs might be a candidate cytokine involved in Treg inhibition.

In this study, we observed that systemic administration of low-dose resiquimod induced a transient (3 hr) upregulation of IFN-α in sera and IL-12, MHC class II, and CD86 expression by pDCs in the RLNs. Thus, pDCs in the circulation, including tumor sites and secondary lymphoid organs, are the initial responsive target cells for resiquimod. We also observed that resiquimod stimulation *in vitro* efficiently increased CD8^+^PDCA-1^+^ DCs and pre-depletion of pDC failed to increase CD8^+^ DCs (Supplementary Figure 3). It has been shown that unlike regular splenic pDCs, TLR ligand-activated pDCs play a critical role in the cross-priming of tumoral-antigens to generate effector CTLs in RLNs [32]. Although we have not directly examined function of resiquimod-treated pDCs, it is possible that resiquimod-stimulated CD8^+^ pDCs may contribute to cross-priming. However, pDCs licensed by TLR ligands alone fail to induce efficient CTLs and cooperate with other RLN cDCs or a subset of XCR1^+^ DCs to promote T-cell recruitment and activation [33–35]. Thus, it is likely that activation of pDCs and cDCs by systemic resiquimod injection and cooperation by both types of licensed DCs amplify T- cell priming against tumor antigens, resulting in efficient induction of CTLs and attenuation of Treg generation. This may explain why low-dose resiquimod efficiently affects the numbers of CD8^+^ T, Tregs, and the CD8/Treg ratio.

We cannot presently rule out the involvement of TAMs or MDSCs in resiquimod action. TILs from SCCVII tumor-bearing mice contained a greater proportion of CD11b^+^ TAMs, which was decreased by resiquimod monotherapy (Supplementary Figure 4B). Recent reports demonstrated that intratumoral injection of a phospholipid-conjugated TLR7 agonist increased the ratio of M1/M2 TAMs in several tumor models [24], and subcutaneous injection of resiquimod in CT26 tumors converted monocytic MDSCs into non-suppressive state [23]. Further studies are required to clarify their involvement.

Nevertheless, our results indicate that systemic administration of low-dose resiquimod as a companion drug with PD-1/PD-L1 blockade therapy offers considerable benefits. Although the effects of intratumoral injection of a TLR7/8 agonist (3M-052/ MEDI9197) alone and in combination with anti-PD-L1 antibody (durvalumab) against solid tumors are being studied in a clinical trial (ClinicalTrials.gov #NCT02556463), intratumoral injection requires the selection of eligible cases and the use of special techniques by medical professionals. Systemic administration is much more convenient and broadly applicable. Once a suitable dose that does not cause adverse effects, as represented by acute proinflammatory cytokine secretion, is determined for clinical use, systemic administration of low-dose resiquimod combined with PD-1/PD-L1 blockade therapy, especially in immunosuppressive tumors, such as HNC, may have a great potential to eradicate tumors. Our results suggest that systemic administration of low-dose resiquimod is useful as a companion drug with PD-1/PD-L1 blockade therapy.

## MATERIALS AND METHODS

### Mice

Female 6–7-week-old BALB/c and C3H mice were purchased from Japan SLC (Hamamatsu, Japan). All mice were maintained under specific pathogen-free conditions at Tokyo Medical and Dental University. All experimental procedures were reviewed and approved by the Animal Care and Use Committee of Tokyo Medical and Dental University (0170344A).

### Tumor cell lines and treatment

Two tumor cell lines, SCCVII (a spontaneously arising squamous cell carcinoma cell line derived from C3H mice, 2 × 10^5^ cells) [10, 25, 26, 29] and Colon 26 (a carcinogen-induced undifferentiated colon carcinoma cell line originated from BALB/c mice, 5 × 10^5^ cells) [29, 36, 37] were used. For induction of PD-L1 *in vitro*, cells were cultured in the presence or absence of IFN-γ (10 ng/ml, BD Biosciences, San Diego, CA) for 72 hr. Cultured tumor cells were freeze-stocked at the same time, and cells that had been cultured for 5–7 days were used for tumor inoculation. Tumor cells were injected subcutaneously (s.c.) into the shaved right flank of syngeneic mice, and tumor size was measured as described previously [10, 29]. Resiquimod was synthesized with ≥98% purity by a contract research organization and provided in lyophilized form. Comparable activity with R848 purchased from InvivoGen (San Diego, CA) was validated by IFN-α, IL-12 and TNF-α secretion using mouse and human pan-DC fractions by ELISA. In most experiments, unless otherwise noted, treatment with either resiquimod (1.7 μg/mouse) or anti-PD-L1 mAb (MIH5, rat IgG2a, 200 μg/mouse) [38], or with both was started at day 0 for Colon 26 and day 7 for SCCVII by intraperitoneal (i.p.) injection twice per week until day 17.

### Isolation of TILs and LN cells

To isolate TILs, resected tumor masses were minced and digested with collagenase I, hyaluronidase, and DNase as described previously [39]. Then alive cells were separated using a lymphocyte separation medium. RLNs were collected from the tumor-inoculated side of inguinal and axillary LNs. For analysis of DCs, RLN cells were isolated using type I collagenase as described previously [40].

### Monoclonal antibodies and flow cytometry

Monoclonal antibodies (mAbs) against CD3 (17A2, rat IgG2b), CD4 (GK1.5, rat IgG2b), CD8 (53-6.72, rat IgG1), CD45 (30-F11, rat IgG2b), IFN-γ (XMG1.2, rat IgG1), Foxp3 (FJK-165, rat IgG2a), CD11b (M1/70, rat IgG2b), CD11c (N418, Armenian hamster IgG), PDCA-1 (eBio927, rat IgG2b), CD86 (PO3.1, rat IgG2b), MHC class II (M5/114, rat IgG2b), PD-L1 (MIH5, rat IgG2a), IL-12/IL-23 p40 (C17.8, rat IgG2a), and Ki-67 (Sola15, rat IgG2a) were used. All FITC-, PE-, APC-, PerCP-Cy5.5-, PE-Cy7-, APC-eFluor780-, V450-, and Brilliant Violet 510-conjugated mAbs were obtained from eBioscience (San Diego, CA), BD-Biosciences or Biolegend (San Diego, CA). Non-specific binding via FcγR and multicolor cell staining for cell surface and intracellular molecules was performed as described previously [10]. Stained cells were analyzed using FACSCalibur or FACSVerse (BD Biosciences) with Cell Quest Pro or FACSuite software. All data were analyzed using FlowJo software (Tree Star, Ashland, OR).

### Assessment of serum proinflammatory cytokines and activation status of DCs after resiquimod injection

Cultured SCCVII cells were 40-Gy irradiated to stop cell growth. Intact or irradiated-SCCVII tumor- (1 × 10^6^ cells/site) inoculated mice received i.p. administration of resiquimod (1.7 μg/mouse); then, blood and RLNs (inguinal and axillary LNs) were collected at the indicated time points. Serum cytokines (IFN-α, IL-1β, IL-6, and TNF-α) were measured using ELISA kits according to the manufacture’s protocol (eBioscience). For measurement of intracellular IL-12, cells were cultured in the presence of brefeldin A for 3 hr.

### Immunohistochemistry

Cryostat sections (6 μm) of tumor tissue were stained with anti- PD-L1 (MIH6, rat IgG2a), and enzymatic immunohistochemistry was performed as described previously [38].

### Statistical analysis

Statistical analyses were performed using the Mann–Whitney *U*-test or the Mann–Whitney *U*-test with Bonferroni correction. Statistical significance was set at *p* < 0.05.

## SUPPLEMENTARY MATERIALS FIGURES



## Abbreviations

APC: antigen-presenting cell
cDC: conventional dendritic cell
CD8/Treg: effector CD8^+^ T cells/Tregs
CTL: cytotoxic T lymphocyte
DC: dendritic cells
HNC: head and neck cancer
MDSC: myeloid-derived suppressor cell
pDC: plasmacytoid dendritic cell
RLN: regional lymph node
SCC: squamous cell carcinoma
TAM: tumor-associated macrophage
Tcon: conventional CD4^+^ T cell
TIL: tumor-infiltrating lymphocyte
TLR: Toll-like receptor
TME: tumor microenvironment
Treg: regulatory T cell
