# Supplementary material for: Systemic administration of a TLR7 agonist attenuates regulatory T cells by dendritic cell modification and overcomes resistance to PD-L1 blockade therapy
Source: Oncotarget. 2018 Jan 27;9(17):13301–12. doi: 10.18632/oncotarget.24327 (PMC5862579; doi:10.18632/oncotarget.24327)
Supplement: Supplementary file 1 [file oncotarget-09-13301-s001.pdf]

Systemic administration of a TLR7 agonist attenuates regulatory T cells by dendritic cell modification and overcomes resistance to PD-L1 blockade therapy

SUPPLEMENTARY MATERIALS

Stimulation of DCs by resiquimod *in vitro*

In a preliminary experiment, a suitable dose of resiquimod that induce activation of DCs *in vitro* was selected. Non-depleted- and pDC-depleted splenocytes from C3H mice were stimulated in the presence or absence of resiquimod (0.1 μg/ml) and expression of IL-12 on PDCA-1$^-$CD11c$^+$ cDCs was analyzed at 12 hr. For depleting pDCs, negative selection was performed by using anti-PDCA-1 MicroBeads (Miltenyi Biotec, San Diego, CA).

Generation of tregs by co-culture with Ag-pulsed DCs and DO11.10 CD4$^+$ T cells

DCs were isolated from BALB/c splenocytes by using Pan DC MicroBeads (Miltenyi Biotec). Isolated DCs (2 × 10^5 cells/well) were pre-cultured in the presence of OVA (10 μg/ml) and TNF-α (10 ng/ml) with or without resiquimod (0.1 μg/ml) in a 48 well plate for 18 hr. DO11.10 TCR CD4$^+$ T cells from RAG2$^{-/-}$ DO11.10 mice were negatively isolated using a BD IMag-DM system (BD Biosciences), and then isolated cells were labeled with violet tracker (CellTrace Violet Cell proliferation Kit, Invitrogen, Carlsbad, CA). Violet labeled DO11.10 CD4$^+$ T cells (1 × 10^6 cells/well) were added to the well of OVA-pulsed DCs, and co-cultured for 3 days. Cultured cells were stained and analyzed by flow cytometry.

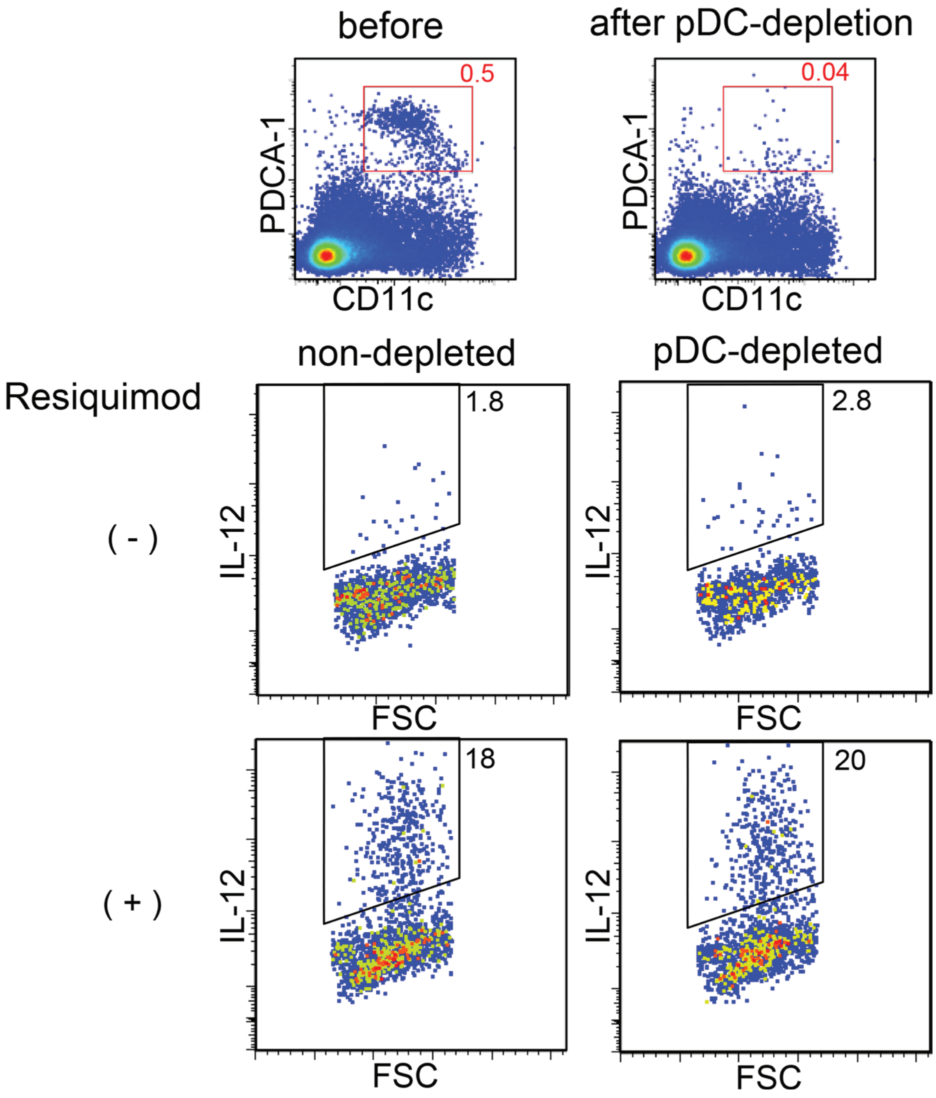

Supplementary Figure 1: Non-depleted and pDC-depleted splenic cDCs comparably induce IL-12 expression after resiquimod stimulation. Non-depleted and pDC-depleted splenocytes were cultured for 12 hr with or without resiquimod. IL-12 expression was measured. Upper two panels show CD11c and PDCA-1 expression in whole and pDC-depleted splenocytes before culture. An electronic gate was placed on PDCA-1$^-$CD11c$^+$ cDCs, and then IL-12 expression and forward scatter (FSC) profiles are displayed as dotted plots. Values are the percentages of IL-12$^+$ cells.

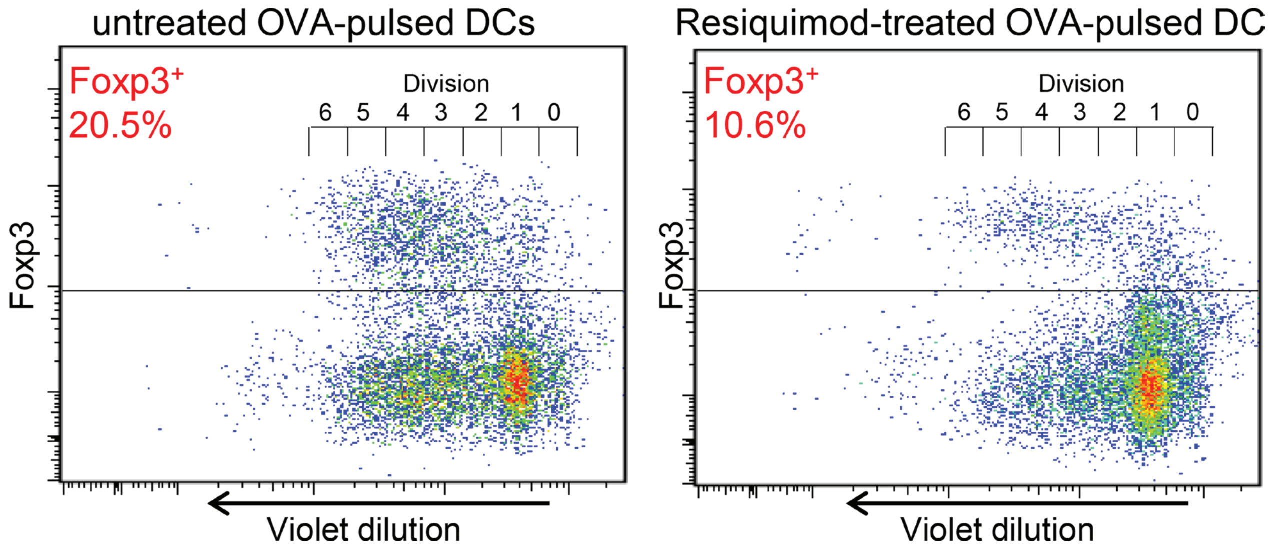

Supplementary Figure 2: Resiquimod-treated DCs inhibit generation of induced Tregs. Violet-labeled DO11.10 CD4+ T cells were co-cultured with untreated or resiquimod-treated OVA-pulsed mature splenic DCs as described in Supplementary Materials and Methods. Cells were stained and analyzed after 3 day culture. An electronic gate was placed on CD4+CD3+ alive lymphocytes and then Foxp3 expression and violet dilution are displayed as dotted plots. Values are the percentages of Foxp3+ cells within CD4+ T cells.

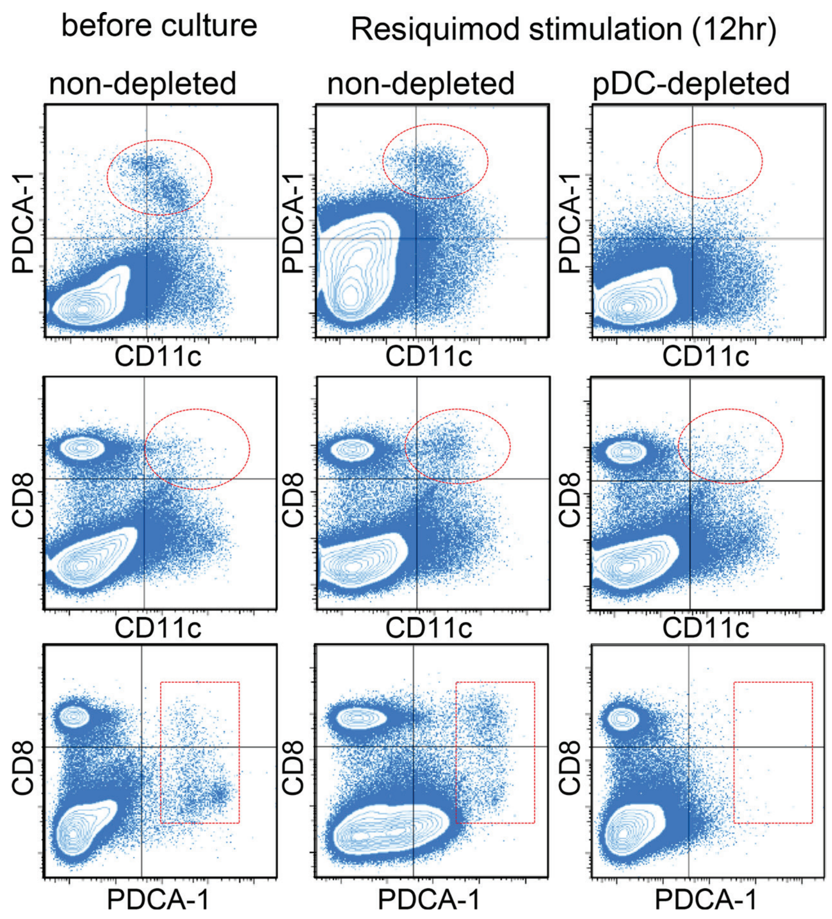

Supplementary Figure 3: Resiquimod stimulation increases CD8+PDCA-1+ DCs. Non-depleted and pDC-depleted splenocytes were cultured in the presence of resiquimod (0.1 μg/ml) for 12 hr. Cells were stained and analyzed by flow cytometry. Expression profiles of CD11c, PDCA-1, and CD8 in alive cells are displayed as dotted plots.

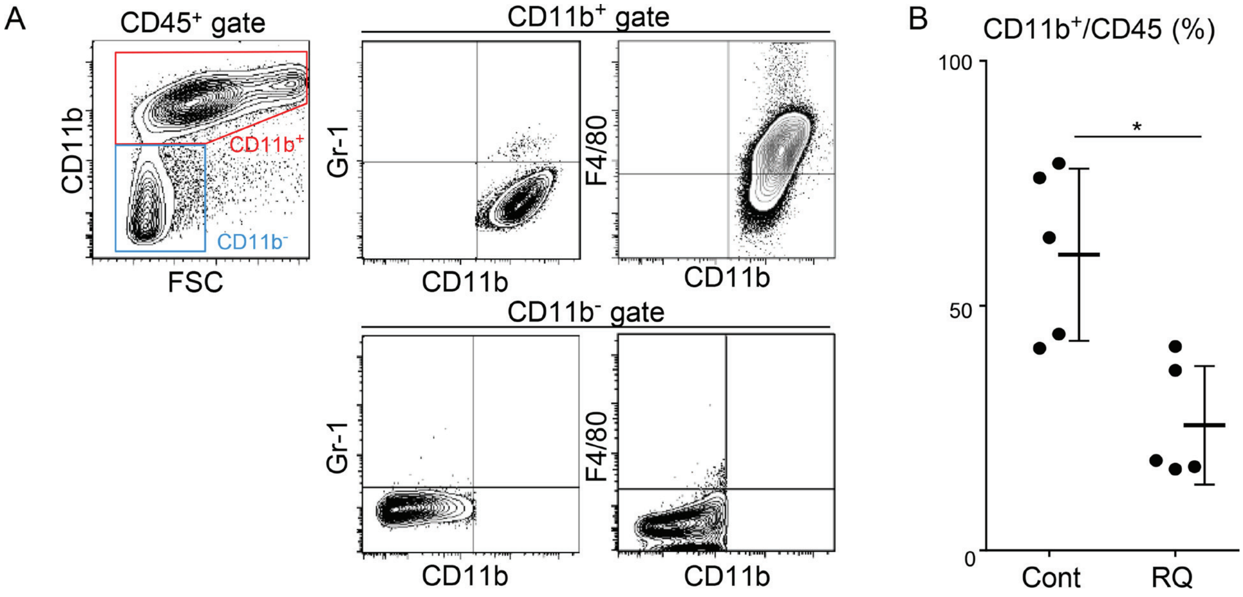

Supplementary Figure 4: CD11b⁺ cells are Gr-1-negative and F4/80-positive, and resiquimod treatment decreases the proportion of CD11b⁺ cells. SCCVII tumor cells were inoculated and mice were treated as described in Figure 2. TIL fractions at day 19 were stained with FITC-anti-CD45, PE-anti-CD11b, APC-anti-F4/80 and V450-anti-Gr-1 mAbs. Flow cytometric profiles in the indicated cell gate are presented as contour plots in (**A**). The proportion of CD11b⁺ cells within CD45⁺ cells was analyzed in (**B**). The bars show the mean values ± SD. *Statistically different ($p < 0.05$).
